# Supplementary figures and images for: Impact of the COVID-19 pandemic on the provision and uptake of services for the prevention of mother-to-child transmission of HIV in Zimbabwe
Source: PLOS Glob Public Health. 2023 Aug 14;3(8):e0002296. doi: 10.1371/journal.pgph.0002296 (PMC10424857; doi:10.1371/journal.pgph.0002296)

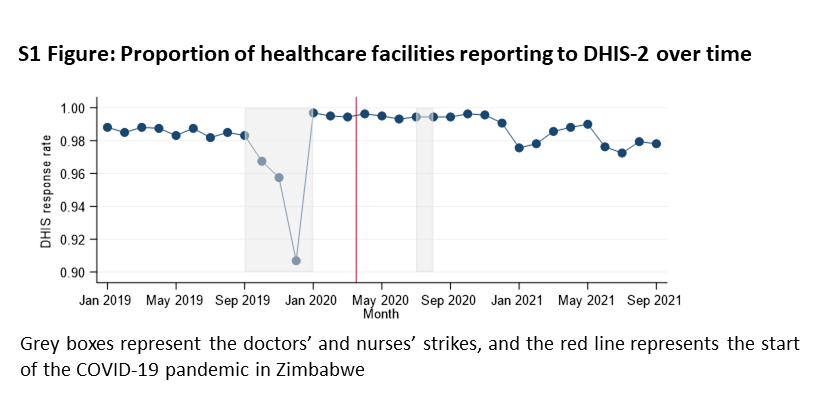

Supplement: S1 Fig — (TIF) [file pgph.0002296.s001.tif]

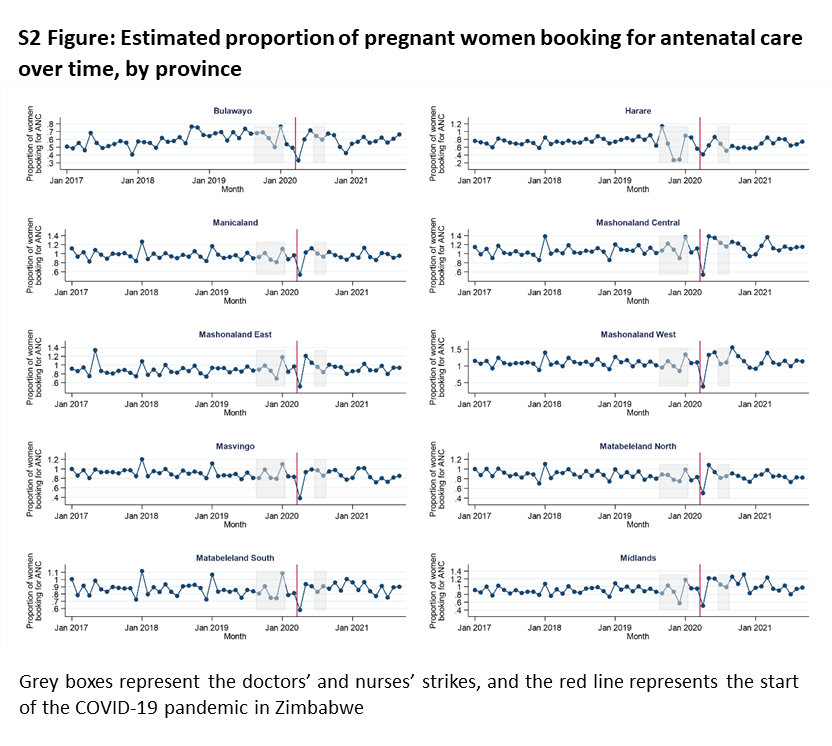

Supplement: S2 Fig — (TIF) [file pgph.0002296.s002.tif]

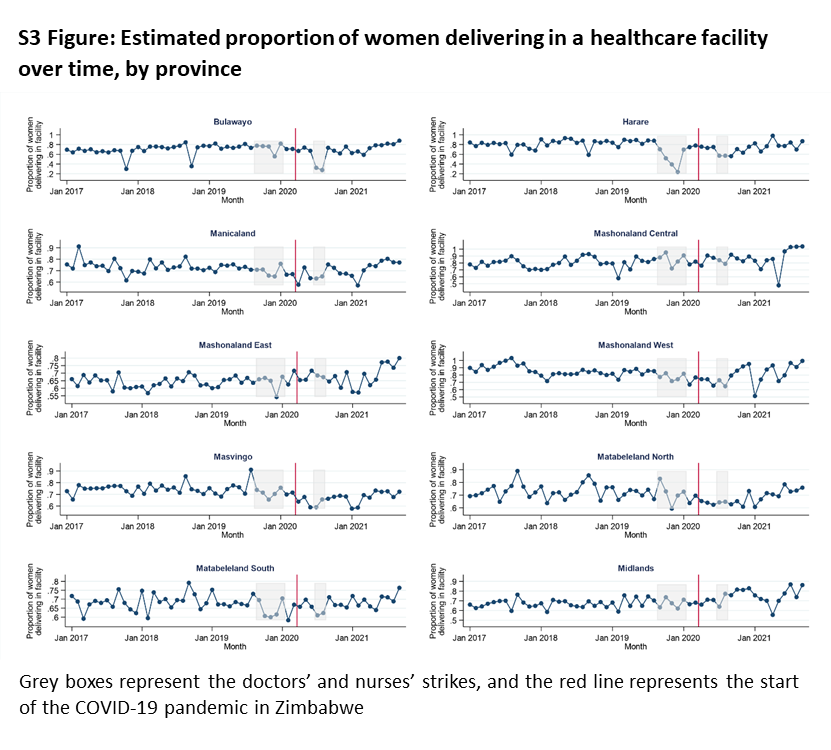

Supplement: S3 Fig — (TIF) [file pgph.0002296.s003.tif]

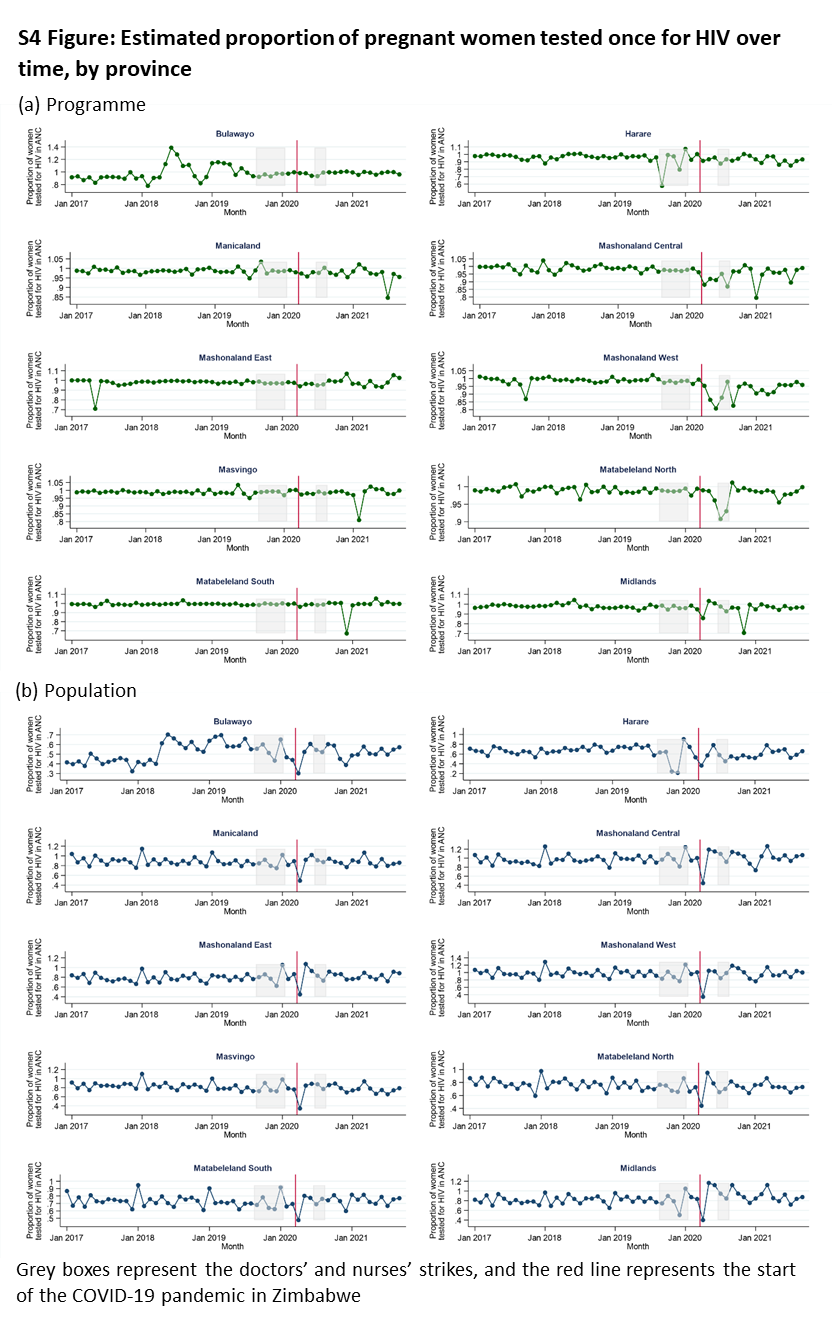

Supplement: S4 Fig — (TIF) [file pgph.0002296.s004.tif]

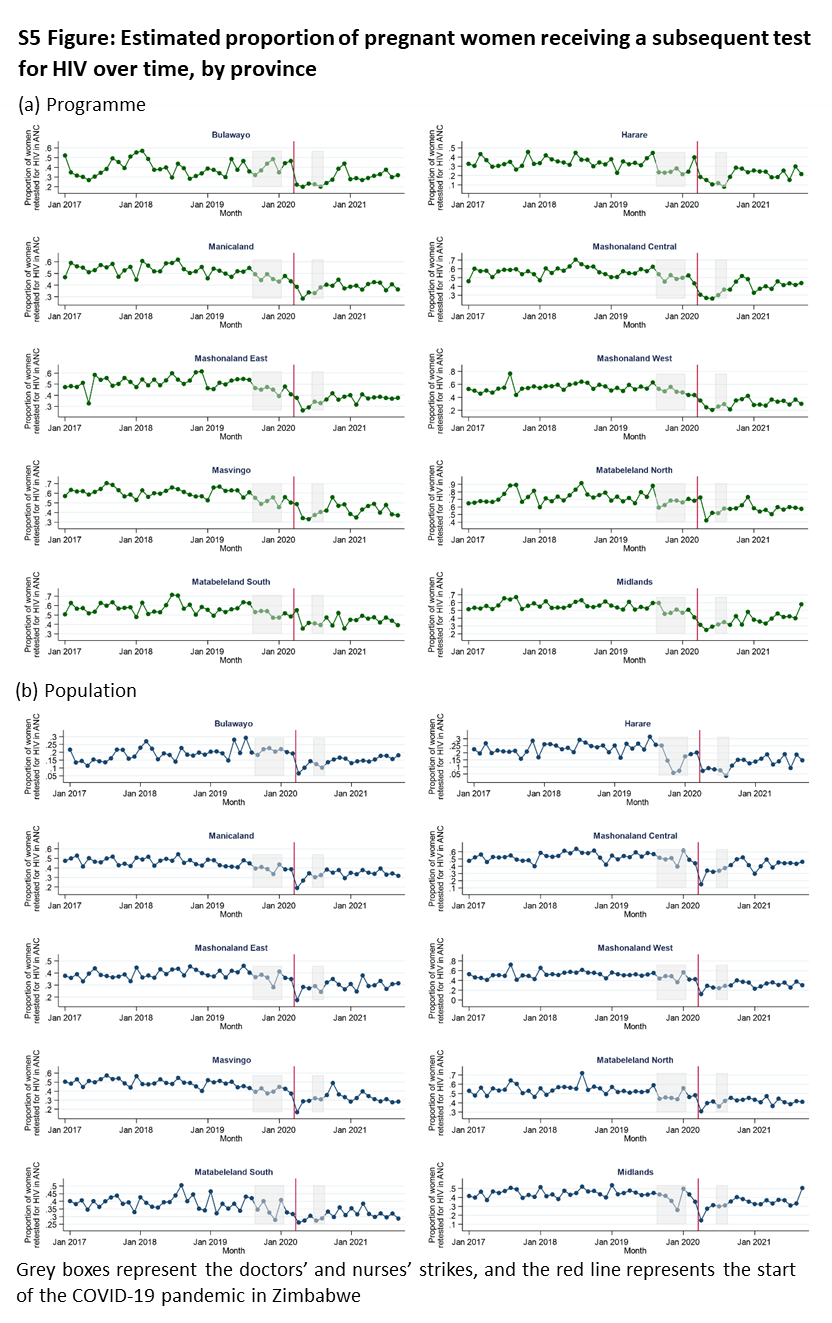

Supplement: S5 Fig — (TIF) [file pgph.0002296.s005.tif]

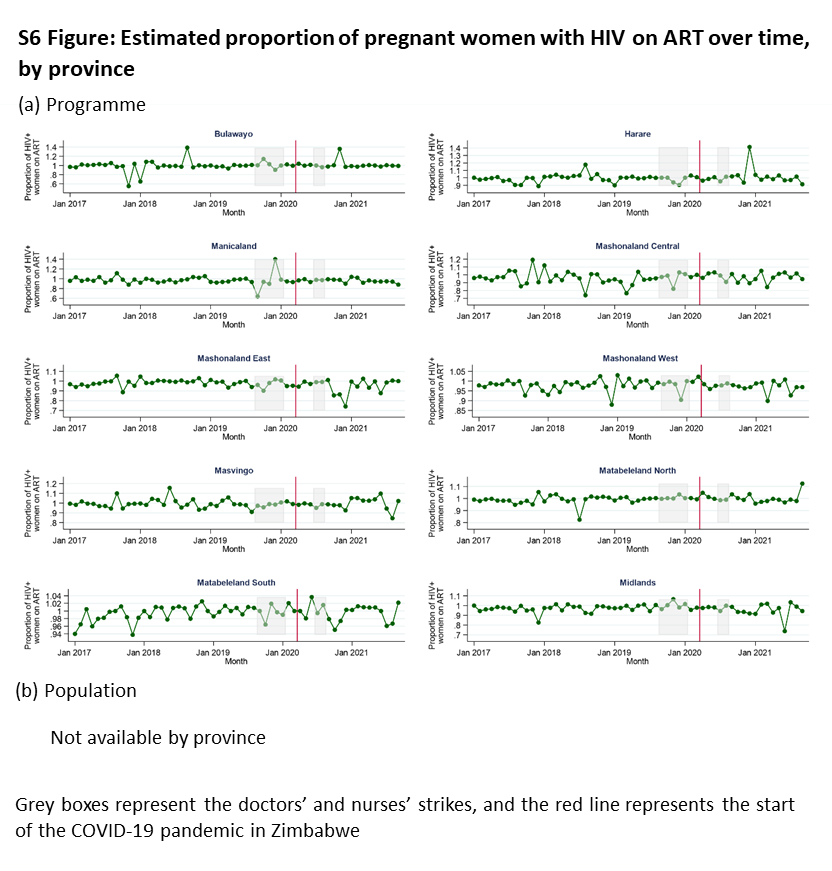

Supplement: S6 Fig — (TIF) [file pgph.0002296.s006.tif]

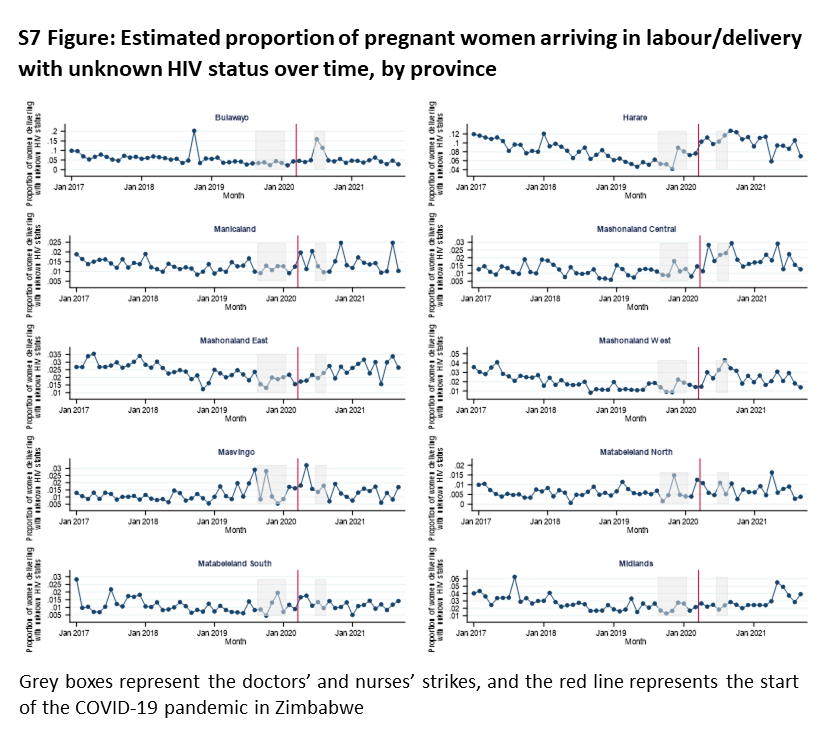

Supplement: S7 Fig — (TIF) [file pgph.0002296.s007.tif]

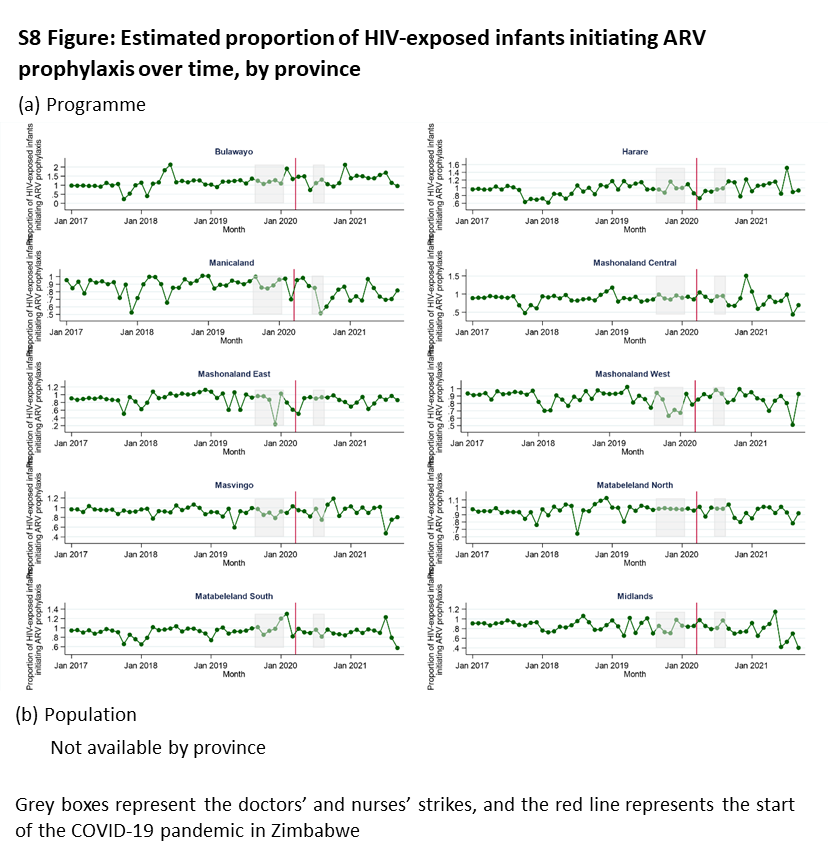

Supplement: S8 Fig — (TIF) [file pgph.0002296.s008.tif]

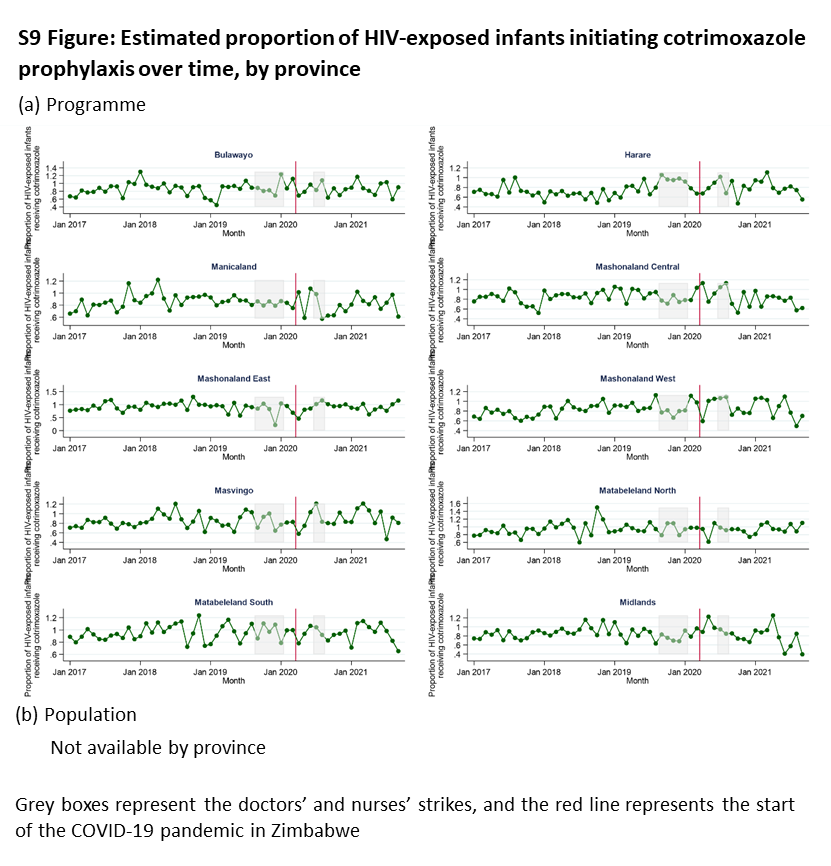

Supplement: S9 Fig — (TIF) [file pgph.0002296.s009.tif]

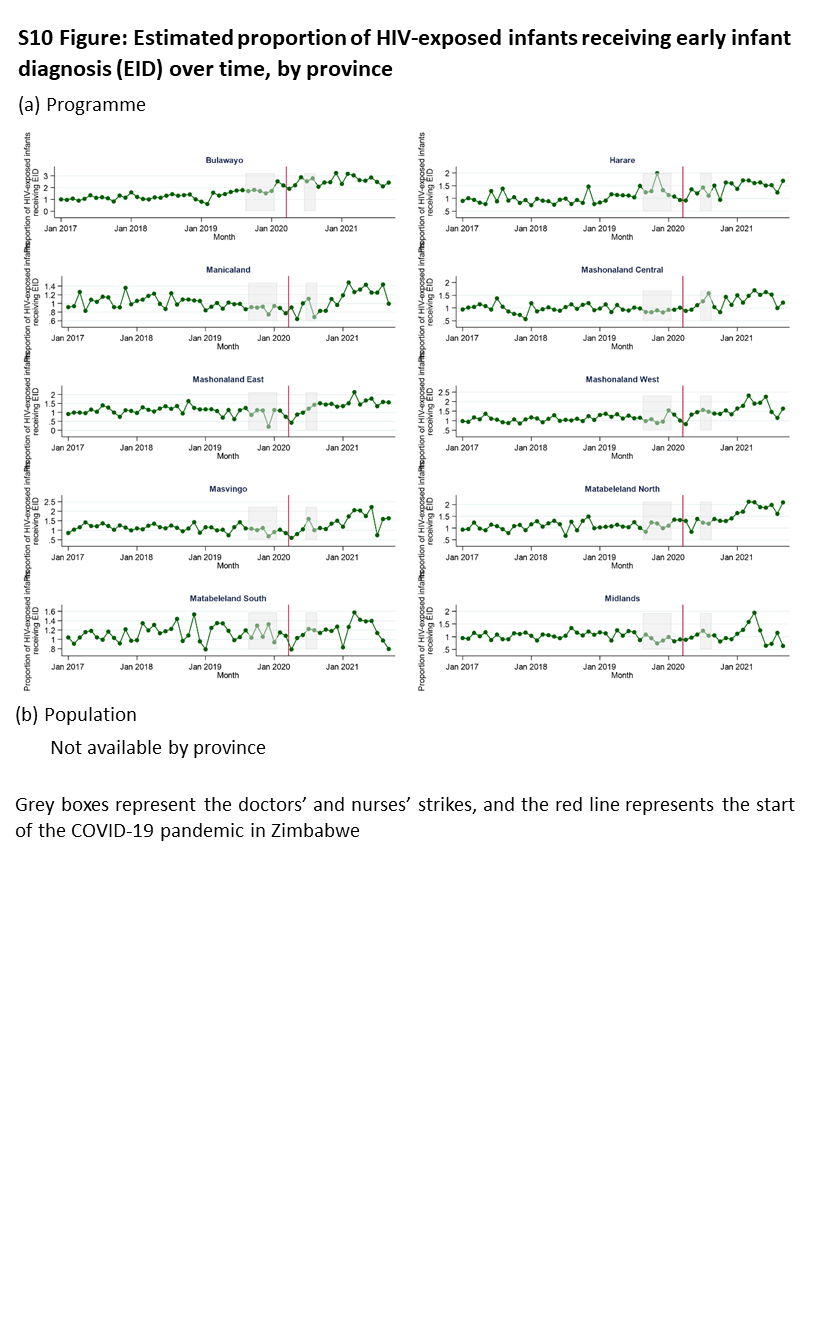

Supplement: S10 Fig — (TIF) [file pgph.0002296.s010.tif]

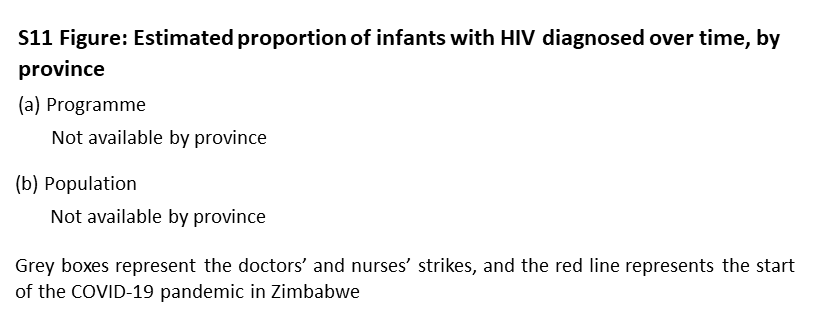

Supplement: S11 Fig — (TIF) [file pgph.0002296.s011.tif]

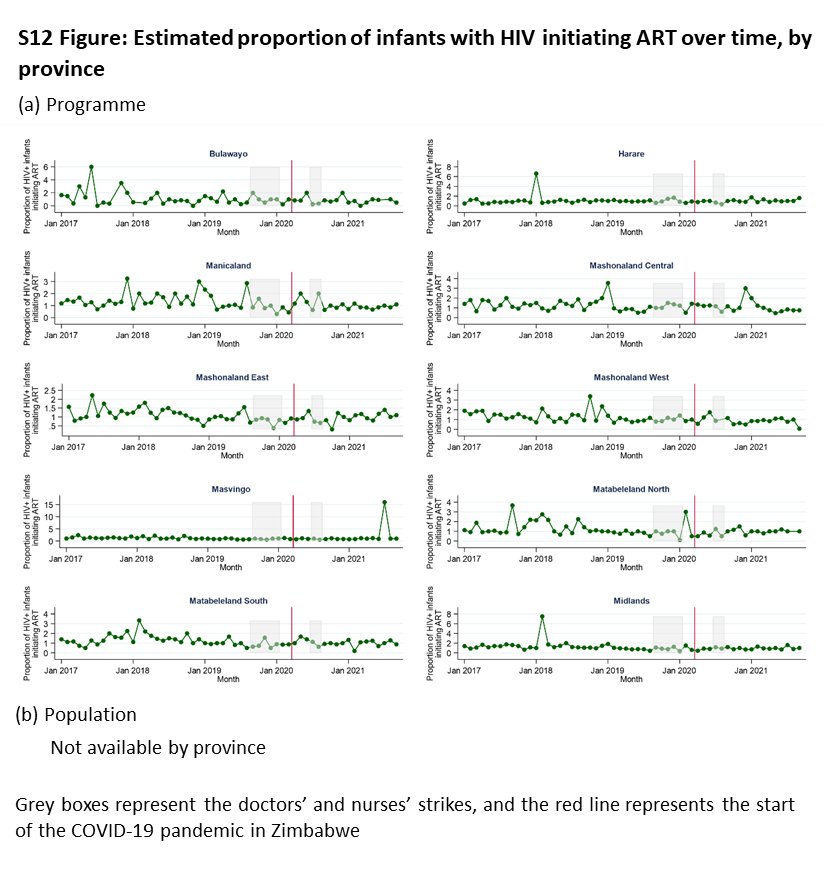

Supplement: S12 Fig — (TIF) [file pgph.0002296.s012.tif]
