# Supplementary material for: Impact of the COVID-19 pandemic on the provision and uptake of services for the prevention of mother-to-child transmission of HIV in Zimbabwe
Source: PLOS Glob Public Health. 2023 Aug 14;3(8):e0002296. doi: 10.1371/journal.pgph.0002296 (PMC10424857; doi:10.1371/journal.pgph.0002296)
Supplement: S1 Table — (DOCX) [file pgph.0002296.s013.docx]

## S1 Table: Results of sensitivity analyses

| **Indicator** | | | **Programme/ population** | **Sensitivity analysis 1:**  **Exclude both strikes** | | | **Sensitivity analysis 2:**  **Include both strikes** | | |
| --- | --- | --- | --- | --- | --- | --- | --- | --- | --- |
|  |  |  |  | **Pre-COVID-19** | **During-COVID-19** | **p** | **Pre-COVID-19** | **During-COVID-19** | **p** |
| **Antenatal care** | 1 | Estimated proportion of pregnant women booking for ANC | Population | 91%  (87%, 94%) | 91%  (86%, 96%) | 0.96 | 90%  (87%, 94%) | 91%  (86%, 96%) | 0.91 |
|  | 2 | Estimated proportion of women delivering in healthcare facility | Population | 74%  (73%, 76%) | 74%  (72%, 76%) | 0.62 | 74%  (72%, 75%) | 73%  (70%, 75%) | 0.52 |
| **Maternal HIV care in antenatal care** | 3 | Estimated proportion of pregnant women tested for HIV | Programme | 98%  (98%, 99%) | 96%  (95%, 96%) | <0.001 | 98%  (97%, 99%) | 96%  (95%, 96%) | <0.001 |
|  |  |  | Population | 81%  (78%, 85%) | 80%  (76%, 85%) | 0.66 | 81%  (78%, 84%) | 80%  (76%, 85%) | 0.70 |
|  | 4 | Estimated proportion of pregnant women retested for HIV | Programme | 53%  (52%, 54%) | 36%  (35%, 38%) | <0.001 | 52%  (51%, 54%) | 36%  (34%, 38%) | <0.001 |
|  |  |  | Population | 42%  (41%, 43%) | 30%  (28%, 31%) | <0.001 | 41%  (40%, 43%) | 29%  (28%, 31%) | <0.001 |
|  | 5 | Estimated proportion of pregnant women with HIV on ART | Programme | 98%  (98%, 99%) | 97%  (97%, 98%) | 0.25 | 98%  (97%, 99%) | 98%  (97%, 98%) | 0.33 |
|  |  |  | Population | 86%  (83%, 89%) | 80%  (75%, 85%) | 0.045 | 86%  (83%, 89%) | 80%  (76%, 85%) | 0.07 |
|  | 6 | Estimated proportion of women delivering with unknown HIV status | Programme | 2.9%  (2.8%, 3.1%) | 3.3%  (3.0%, 3.6%) | 0.030 | 2.8%  (2.7%, 3.0%) | 3.3%  (3.0%, 3.6%) | 0.004 |
| **Care for HIV- exposed infants** | 7 | Estimated proportion of HIV-exposed infants initiating ARV prophylaxis | Programme | 94%  (91%, 96%) | 87%  (84%, 90%) | 0.001 | 92%  (89%, 95%) | 87%  (84%, 91%) | 0.024 |
|  |  |  | Population | 75%  (74%, 78%) | 69%  (66%, 71%) | <0.001 | 75%  (72%, 77%) | 68% | 0.001 |
|  | 8 | Estimated proportion of HIV-exposed infants receiving CTX | Programme | 85%  (82%, 87%) | 82%  (78%, 86%) | 0.28 | 84%  (82%, 87%) | 83%  (79%, 87%) | 0.76 |
|  |  |  | Population | 69%  (67%, 70%) | 65%  (62%, 68%) | 0.030 | 68%  (66%, 70%) | 65%  (62%, 67%) | 0.034 |
|  | 9 | Estimated proportion of HIV-exposed infants receiving EID | Programme | - | - | - | 106%  (103%, 109%) | 103%  (92%, 116%) | 0.664 |
|  |  |  | Population | - | - | - | 86%  (83%, 89%) | 79%  (70%, 88%) | 0.153 |
| **Care for infants with HIV** | 10 | Estimated proportion of infants with HIV diagnosed | Population | 27%  (25%, 28%) | 19%  (17%, 21%) | <0.001 | 26%  (25%, 28%) | 18%  (17%, 20%) | <0.001 |
|  | 11 | Estimated proportion of infants with HIV on ART | Programme | 117%  (110%, 125%) | 91%  (80%, 102%) | <0.001 | 114%  (107%, 121%) | 90%  (80%, 101%) | <0.001 |
|  |  |  | Population | 31%  (29%, 33%) | 17%  (15%, 20%) | <0.001 | 30%  (28%, 32%) | 16%  (14%, 19%) | <0.001 |
