# Supplementary material for: Impact of the COVID-19 pandemic on the provision and uptake of services for the prevention of mother-to-child transmission of HIV in Zimbabwe
Source: PLOS Glob Public Health. 2023 Aug 14;3(8):e0002296. doi: 10.1371/journal.pgph.0002296 (PMC10424857; doi:10.1371/journal.pgph.0002296)
Supplement: S2 Table — (DOCX) [file pgph.0002296.s014.docx]

## S2 Table: Results using data from the full time period for indicators where there was an ongoing trend prior to the start of the pandemic

| **Indicator** | | | **Programme/ population** | **Pre-COVID-19** | | **During-COVID-19** | **p** | **Change pre-COVID-19**  **(per month)** | **Change at start of pandemic** | **Change during-COVID-19 (per month)** |
| --- | --- | --- | --- | --- | --- | --- | --- | --- | --- | --- |
|  |  |  |  | *Estimate (95% CI)* | | |  | *Relative reduction (95% CI), p-value* | | |
| **Maternal HIV care in antenatal care** | 6 | Estimated proportion of women delivering with unknown HIV status | Programme | 2.9%  (2.8%, 3.1%) | 3.3%  (3.1%, 3.6%) | | 0.017 | 0.98  (0.98, 0.99), p<0.001 | 1.74  (1.54, 1.97), p<0.001 | 0.99  (0.98, 1.00), p=0.08 |
| **Care for HIV- exposed infants** | 8 | Estimated proportion of HIV-exposed infants receiving CTX | Programme | 85%  (82%, 87%) | 83%  (80%. 87%) | | 0.597 | 1.00  (1.00, 1.01), p=0.01 | 0.98  (0.88, 1.08), p=0.69 | 0.99  (0.98, 1.00), p=0.06 |
|  |  |  | Population | 69%  (67%, 70%) | 65%  (62%, 67%) | | 0.012 | 1.00  (1.00, 1.01), p=0.011 | 0.85  (0.77, 0.93), p<0.001 | 1.00  (1.00, 1.01), p=0.21 |
| **Care for infants with HIV** | 11 | Estimated proportion of infants with HIV on ART | Programme | 117%  (110%, 124%) | 90%  (80%, 101%) | | <0.001 | 0.99  (0.99, 1.00), p=0.002 | 0.98  (0.76, 1.25), p=0.856 | 0.99  (0.97, 1.02), p=0.62 |
|  |  |  | Population | 31%  (29%, 33%) | 16%  (14%, 19%) | | <0.001 | 0.99  (0.98, 0.99), p<0.001 | 0.69  (0.52, 0.91), p=0.009 | 1.00  (0.98, 1.02), p=0.99 |
