# Supplementary material for: Impact of the COVID-19 pandemic on the provision and uptake of services for the prevention of mother-to-child transmission of HIV in Zimbabwe
Source: PLOS Glob Public Health. 2023 Aug 14;3(8):e0002296. doi: 10.1371/journal.pgph.0002296 (PMC10424857; doi:10.1371/journal.pgph.0002296)
Supplement: S1 File — (PDF) [file pgph.0002296.s015.pdf]

MINISTRY OF HEALTH AND CHILD CARE ZIMBABWE

PMTCT , HIVST, HTS, HIV/TB, OI/ART, PrEP, PEP, STI/HIV, SEXUAL VIOLENCE &

VMMC MONTHLY PROGRESS RETURN FORM

REPORTING UNIT:							CODE:
PROVINCE:		CODE:		DISTRICT:			CODE:
TELEPHONE:		E-MAIL:					
START DATE	Day:	Month:	Year:	END DATE	Day:	Month:	Year:

Report timeliness:

The reporting deadlines for all the monthly progress **return** forms are as **follows**;

- **Health** Facility to District (Paper based report): By the 7th
- **District** to Province (Electronic system - DHIS2): By the 21st
- **Province** to Head Office (Electronic system - DHIS2): By the 28th

Site Level	District Level
Prepared By: ....<br>Date------------/----------/---------------	**Received By...**<br>**Date-----------/---------/-----------**
**Checked By...**<br>**Date ------------/----------/--------------**	**Checked by...**<br>**Date ----------/-------/-------**
**Checked By..**<br>**Sent by ……..**<br>**Date --------/-----------/---------**	

Revised V4: October 2018

Instructions on the use of this form:

1. Each department offering a service should have a register for documenting the service or commodity offered.
2. All monthly progress return forms should be completed in ink.
3. All sections should be completed legibly showing that data were checked and verified at different levels within the facility.
4. Where a service that is supposed to be offered at the facility or within a department has not been offered, indicate with zeros (0).
5. Where a service is not available or irrelevant, indicate with not applicable (N/A) across data elements.
6. For the cover page under timeliness section, at a facility where there is one health worker, only complete the '***prepared by and sent by fields*** "and leave the" ***checked by*** "field blank. Indicate in the notes section located at the back of the form, the reason/s why "***checked by*** "fields are not completed.
7. All departments/service delivery points should submit their completed monthly progress return forms to Health Information department on time, and ensure that correct figures for their department's contribution are added to the consolidated facility figures.
8. All service delivery points should remain with a copy of the monthly progress return form that they submit to Health Information Department for their file.
9. Health Information department should capture all monthly progress return forms received from the different service delivery points/departments into DHIS2.
10. A copy of the Monthly Progress Return Forms from the different departments must be attached to the consolidated form for the facility and filed in the Health Information department.
11. On the HIV Testing services section, include the PMTCT testing and the exposed infants who received EID results during the reporting period.

Revised V4: October 2018

A. PMTCT

I. HIV Testing Services in ANC	Data Source	10-14 years	15-19 years	20-24 years	25-29 years	30-34 years	35-39 years	40-44 years	45-49 years	50 years and above	Total
P1. Pregnant women booking for ANC contact	ANC										
P2. Pregnant women booking for first ANC contact with known HIV positive result	ANC										
P3. Pregnant women HIV tested for the First time in ANC and received results	HTS										
P4. Pregnant women newly testing HIV Positive in ANC	HTS										
P5. Pregnant women retested for HIV in ANC and received results	HTS										
P6. Pregnant women testing HIV Positive at retest in ANC	HTS										
P7. HIV Positive pregnant women screened for TB in ANC	ANC										
P8. Male partners with a known HIV status *(Include those already HIV+ and those tested in ANC)*	ANC										
**II. Maternal Syphilis and ART in ANC**	**Data Source**	**10-14 years**	**15-19 years**	**20-24 years**	**25-29 years**	**30-34 years**	**35-39 years**	**40-44 years**	**45-49 years**	**50 years and above**	**Total**
P9. Pregnant women booking for ANC already on ART	ANC										
P10. HIV positive pregnant women initiated on ART in ANC < 32 weeks GA	ART										
P11. HIV positive pregnant women initiated on ART in ANC ≥ 32 weeks GA	ART										
P12. HIV positive pregnant women with a viral load ≥ 1000 copies/ml											
P13. Pregnant women tested for Syphilis for the first time in ANC	ANC										
P14. Pregnant women testing Syhilis Positive in ANC	ANC										
P15. Pregnant women who received treatment for Syphilis (at least one dose)	ANC										
**III. HIV Testing Services & ART in Labour and Delivery (LD)**	**Data Source**	**10-14 years**	**15-19 years**	**20-24 years**	**25-29 years**	**30-34 years**	**35-39 years**	**40-44 years**	**45-49 years**	**50 years and above**	**Total**
P16. Pregnant women arriving in LD with unknown HIV status	DEL										
P17. Pregnant women tested in LD for the first time and received results	HTS										
P18. Pregnant women testing HIV positive at first test in LD	HTS										
P19. Pregnant women retested in LD and received results	HTS										
P20. Pregnant women testing HIV positive at retest in LD	HTS										
P21. HIV positive pregnant women initiated on ART in LD	ART										

A. PMTCT

III. HIV Testing Services & ART in Labour and Delivery (LD)	Data Source	10-14 years	15-19 years	20-24 years	25-29 years	30-34 years	35-39 years	40-44 years	45-49 years	50 years and above	Total
P22. Total Institutional Deliveries	DEL										
P23. Total Deliveries by HIV Positive women *(include BBA, Home deliveries, Institutional deliveries)*	DEL										
**HIV Testing services and ART in Post delivery**	**Data Source**	**10-14 years**	**15-19 years**	**20-24 years**	**25-29 years**	**30-34 years**	**35-39 years**	**40-44 years**	**45-49 years**	**50 years and above**	**Total**
P24. Women arriving with unknown HIV status within 24 months PD	PNC/ MBP										
P25. Women HIV tested for the first time within 24 months PD and received results	HTS										
P26. Women testing HIV positive for the first-time within 24 months PD	HTS										
P27. Women retested within 24 months PD and received results	HTS										
P28. Women testing HIV positive at retesting in PD	HTS										
P29. HIV positive women initiated on ART within 24 months PD	ART										
**V. Infant congenital syphilis and ARV prophylaxis**	**Data Source**	**≤ 72hrs**	**73hrs-24 months**								**Total**
P30. Syphilis Exposed infants treated with single dose of Benzathine penicillin	MBP/ PNC										
P31. HIV exposed infants initiated on AZT + NVP	Inf Disp										
P32. HIV exposed infants initiated on NVP	Inf Disp										
**VI. HIV Testing Services and care for HIV Exposed Infants**	**Data Source**	**≤ 72hrs**	**73 hrs-2 months**	**2-12 months**	**13-24 months**						**Total**
P33. HIV Exposed Infants Initiated on Cotrimoxazole prophylaxis	Inf Disp										
P34. HIV Exposed Infants with EID sample collected (POC /DBS)	CR										
P35.HIV Exposed Infants Testing HIV positive	CR										
P36. Infants initiated on ART	ART										

HIV TESTING SERVICES.

	HIV SELF TEST, Data source: HTS-Self-Test Register)																																		
	FACILITY BASED																	COMMUNITY BASED																	
	15-19 years		20-24 years		25-29 years		30-34 years		35-39 years		40-44 years		45-49 years		50 years and above		TOTAL	15-19 years		20-24 years		25-29 years		30-34 years		35-39 years		40-44 years		45-49 years		50 years and above		TOTAL	GRAND TOTAL
Sex	M	F	M	F	M	F	M	F	M	F	M	F	M	F	M	F		M	F	M	F	M	F	M	F	M	F	M	F	M	F	M	F		
S1. Number of Self-test kits distributed this month. (Primary Distribution)																																			
S2. Number of Self-test kits distributed this month (Secondary distribution, excluding for partners)																																			
S3. Number of Self-test kits distributed this month (Secondary distribution, for partner).																																			
S4. Number of clients tested for the first time (First ever HIV Test) this month.																																			
S5 Number of Clients with Reactive results on the first test this month.																																			
S6 Number of clients retesting for HIV this month.																																			
S7 Number of clients with Reactive results on re-tests this month.																																			
S8 Total number of results shared this month.																																			
S9. Total number of Reactive results (S5+S7) this month.																																			
S10. Total number of Reactive tests confirmed with provider testing.																																			

B.HIV TESTING SERVICES, INCLUDING PMTCT AND TESTING SERVICES OFFERED AT ALL THE OTHER ENTRY POINTS e.g. EID, VMMC, WARDS, EPI ETC. (DATA SOURCE: HTS REGISTER FOR ALL INDICATORS AND EID REGISTER)

AGE	≤2 months		3-12 months		13-24 months		25-59 months		5-9 years		10-14 years		15-19 years		20-24 years		25-29 years		30-34 years		35-39 years		40-44 years		45-49 years		50 years and above		Total
SEX	M	F	M	F	M	F	M	F	M	F	M	F	M	F	M	F	M	F	M	F	M	F	M	F	M	F	M	F	
B1. Number of clients receiving First ever test for HIV this month																													
B2.Number of clients who tested HIV positive for the first ever test this month.																													
B3. Number of clients who were retested for HIV *(excluding for ART initiation)* this month																													
B4. Number of clients who tested HIV positive *(excluding for ART initiation)* during retest this month.																													
B5 Number of clients HIV tested who received results and post-test counselled at the Facility this month (PITC and CITC).																													
B6 Number of clients tested HIV positiveat the Facility this month (PITC and CITC).																													
B7 Number of clients HIV tested who received results and post-test counselled (FACILITY index case testing) this month.																													
B8 Number of clients tested HIV positive this month (FACILITY index case testing)																													
B9. Number of HIV tested who received results and post-test counselled in the COMMUNITY this month.																													
B10. Number of clients tested HIV positive in the COMMUNITY, this month.																													
B11. Number of clients HIV tested who received results and post-test counselled (COMMUNITY index case testing) this month.																													
B12. Number of clients tested HIV positive this month (COMMUNITY index case testing).																													
B13 Total number of clients HIV tested who received results and post-test counselled this month. (B5+B7+B9+B11).																													
B14.Total Number of clients tested HIV positive this month (B2+B4)/(B6+B8+B10+B12). Excluding for ART initiation.																													
B15.Total Number of HIV positive clients tested for recent infection this month.																													
B16.Total Number of HIV positive clients with a recent infection this month.																													
B17 Total number of positive clients linked to OI/ART services this month.																													

ORAL PRE – EXPOSURE PROPHYLAXIS (PrEP) REPORTING ***(DATA SOURCE: PrEP REGISTER)***

AGE (YEARS)	10 - 14		15 - 19		20 - 24		25 - 29		30 - 34		35 - 39		40 - 44		45 - 49		≥50		Total
SEX	M	F	M	F	M	F	M	F	M	F	M	F	M	F	M	F	M	F	
Pr1. Total Number of individuals who have been newly enrolled on oral antiretroviral pre-exposure prophylaxis to prevent HIV infection this month (Prep register)																			
Pr2. Total Number of oral antiretroviral pre-exposure prophylaxis clients continuing PrEP this month (Prep register)																			
Pr3. Total Number of clients experiencing moderate and severe adverse events following oral antiretroviral pre-exposure prophylaxis (Prep register)																			

C.HIV/TB COLLABORATION

AGE	≤ 2 months		3-12 months		13-24 months		25-59 months		5-9 years		10-14 years		15-19 years		20-24 years		25-29 years		30-34 years		35-39 years		40-44 years		45-49 years		50 years and above		Total
**SEX**	M	F	M	F	M	F	M	F	M	F	M	F	M	F	M	F	M	F	M	F	M	F	M	F	M	F	M	F	
C1.Number of TB patients in care tested for HIV this month. (TB register).																													
C2.Number of TB patients in care who tested HIV positive this month. *(TB register).*																													
C3.Number of PLHIV in care screened for TB during their last visit this month .*(Pre -ART & ART registers)*																													
C4.Number of PLHIV in care screened for TB and had signs of active TB disease (Presumptive Cases) *(Pre -ART & ART registers)*																													
C5.Number of PLHIV in care investigated for active TB disease (Active TB) this month *(Pre -ART & ART register)*																													
C6.Number of PLHIV in care tested positive for active TB disease this month *(Pre -ART & ART registers)*																													
C7 .Number of PLHIV newly enrolled in HIV care with active TB disease. *(Pre-ART).*																													
C8 .Number of PLHIV in care newly diagnosed with active TB disease before ART initiation. *(Pre -ART).*																													
C9. Number PLHIV in care newly initiated on TB treatment this month *(Pre -ART and ART registers).*																													
C10. Number of PLHIV in care with active TB disease started on ART this month. *(ART register)*																													
C11.Number of PLHIV screened for TB and eligible for TB Preventive Therapy (incl IPT) this month. *(Pre -ART & ART registers).*																													
C12.Number of PLHIV newly enrolled in HIV care who were initiated on TB Preventive Therapy (incl IPT) this month. *(IPT register).*																													
C13.Number of PLHIV in care started on TB Preventive Therapy (incl IPT) this month. *(IPT register).*																													
C14.Number of PLHIV in care known to have died whilst on TB Preventive Therapy (incl IPT) this month. *(IPT/Essential Changes registers)*																													
C15.Number of PLHIV in care lost to follow up whilst on TB Preventive Therapy (incl IPT) this month *(IPT/Essential Changes registers).*																													
C16.Number of PLHIV in care on TB Preventive Therapy (incl IPT) who developed adverse events this month.*(IPT/Essential Changes register)*																													
C17.Number of PLHIV in care who stopped TB Preventive Therapy (incl IPT) due to severe adverse events this month**(IPT/Essential Changes registers)*																													
C18.Number of PLHIV in care transferred out on TB Preventive Therapy (incl IPT) this month *(IPT/Essential Changes registers).*																													
C19.Number of PLHIV In care transferring in whilst on TB Preventive Therapy (incl IPT) this month *(IPT/Essential Changes registers).*																													
C20.Number of PLHIV in care on TB Preventive Therapy (incl IPT) developing active TB disease this month. *(IPT Register).*																													
C21 .Number of PLHIV in care who completed TB Preventive Therapy (incl IPT) course this month. *(IPT register).*																													

D.OPPORTUNISTIC INFECTIONS AND ANTIRETROVIRAL THERAPY

TREATMENT AND PROPHYLAXIS / AGE	≤2 months		3-12 months		13-24 months		25-59 months		5-9 years		10-14 years		15-19 years		20-24 years		25-29 years		30-34 years		35-39 years		40-44 years		45-49 years		50 years and above		Total
SEX	M	F	M	F	M	F	M	F	M	F	M	F	M	F	M	F	M	F	M	F	M	F	M	F	M	F	M	F	
D1.Number of newly diagnosed PLHIV registered into care this month. *(Pre-ART)*																													
D2.Number of newly diagnosed PLHIV in care in WHO Stage 1 at registration this month *(Pre-ART)*																													
D3.Number of newly diagnosed PLHIV in care in WHO Stage 2 at registration this month *(Pre -ART)*																													
D4.Number of newly diagnosed PLHIV in care in WHO Stage 3 at registration this month *(Pre -ART)*																													
D5.Number of newly diagnosed PLHIV in care in WHO Stage 4 at registration this month *(Pre -ART)*																													
D6. Total number of PLHIV in care started on CTX prophylaxis (including TB patients) this month. *(Pre –ART & ART register)*.																													
D7.Number of PLHIV in care on CTX who developed adverse events this month *(Essential Changes register)*																													
D8.Number of PLHIV in care on CTX stopping due to severe adverse events *(Essential Changes register)*																													
D9.Total number of PLHIV in care currently receiving CTX prophylaxis (including TB patients). *(Pre -ART & ART registers)*																													
D10. Number of PLHIV in care known to have died before initiation on ART this month *(Essential Changes register +Pre -ART)*																													
D11. Number of PLHIV in care lost to follow up before initiation on ART this month *(Essential Changes register +Pre-ART)*																													
D12. Number of PLHIV in care transferred out before initiation on ART this month *(Essential Changes register +Pre -ART)*																													
D13. Number of PLHIV in care who transferred in before initiation on ART this month. *(Essential Changes register +Pre -ART)*																													
D14.Total number of PLHIV in care currently on Pre - ART this month. (Pre- ART)																													
D15.Number of PLHIV newly diagnosed with Cryptococal Meningitis (CM) and commenced on Fluconazole treatment this month *(Pre –ART,ART and Fluconazole registers)*																													
D16.Number of PLHIV in care with Cryptococal Meningitis on Fluconazole treatment who developed adverse events this month *(Essential Changes register)*																													
D17. Number of PLHIV in care with Cryptococcal Meningitis on Fluconazole treatment who stopped treatment due to severe adverse events this month. *(Essential Changes register)*																													

N.B. Please ensure all adverse events /toxicities due to IPT are notified to the Head of station within 48 hours and the appropriate Adverse Drug Reaction (ADR) forms are compiled and sent to the MCAZ through the institution's Pharmacy Manger.

LABORATORY SERVICES

AGE	≤ 2 months		3-12 months		13-24 months		25-59 months		5-9 years		10-14 years		15-19 years		20-24 years		25-29 years		30-34 years		35-39 years		40-44 years		45-49 years		50 years and above		Total
**SEX**	**M**	**F**	**M**	**F**	**M**	**F**	**M**	**F**	**M**	**F**	**M**	**F**	**M**	**F**	**M**	**F**	**M**	**F**	**M**	**F**	**M**	**F**	**M**	**F**	**M**	**F**	**M**	**F**	
D18 .Number of PLHIV in care on ART who had a sample collected for Viral Load testing this month. *(Viral Load Laboratory Request Form)*																													
D19.Number of PLHIV in care on ART who received Viral Load test results this month. *(Viral Load Laboratory Request Form)*																													
D20.Number of PLHIV in care on ART who received Viral Load test results >1,000 copies/ml this month. *(Viral Load Laboratory Request Form)*																													
D21.Number of PLHIV in care on ART who received Viral Load test results >1,000 copies/ml, who received Enhanced Adherence Counselling this month. *(High Viral Load Form)*																													
D22.Number of PLHIV in care on ART who completed EAC and had second VL Test done this month. *(High Viral Load Form).*																													
D23.Number of PLHIV in care on ART who had high second Viral Load test results >1,000 copies/ml this month. *(High Viral Load Form)*																													
D24.Number of PLHIV in care on First line switching to Second line this month. *(ART register-Essential Changes register)*																													
D25.Number of PLHIV in care on Second line switching to Third line regimen this month *(Essential Changes Register)*																													
D26. Number of PLHIV in care initiated on ART with a CD4 count <100 cell/mm^3 this month. *(ART register)*																													
D27.Number of PLHIV in care initiated on ART with a CD4 count >100 and <350 cell/ mm^3 this month. *(ART register)*																													
D28 .Number of PLHIV in care initiated on ART with a CD4 count >350 to <500 cell/mm^3 this month. *(ART register)*																													
D29.Number of PLHIV in care initiated on ART with a CD4 count >500 cell/mm^3 this month. *(ART register)*																													

ART SUMMARY

AGE	≤2 months		3-12 months		13-24 months		25-59 months		5-9 years		10-14 years		15-19 years		20-24 years		25-29 years		30-34 years		35-39 years		40-44 years		45-49 years		50 years and above		Total
**SEX**	**M**	**F**	**M**	**F**	**M**	**F**	**M**	**F**	**M**	**F**	**M**	**F**	**M**	**F**	**M**	**F**	**M**	**F**	**M**	**F**	**M**	**F**	**M**	**F**	**M**	**F**	**M**	**F**	
D30. Number of PLHIV in care newly enrolled medically eligible and completed counselling . sessions but not yet started on ART this Month. *(Pre -ART)*																													
D31 .Number of PLHIV in care retested for verification of HIV at ART initiation this month. *(ART register)*																													
D32. Number of PLHIV in care newly initiated on first line ART this month. *(ART register)*																													
D33. Number of PLHIV in care who have died whilst on First line regimen this month *(Essential Changes register)*																													
D34. Number of PLHIV lost to follow up whilst on First line regimen this month *(Essential Changes register).*																													
D35. Number of PLHIV in care who stopped treatment whilst on First line regimen this month *(Essential Changes register)*																													
D36. Number of PLHIV in care who transferred out whilst on First line regimen this month. *(Essential Changes register)*																													
D37. Number of PLHIV in care who transferred in whilst on First line regimen this month *(Essential Changes register)*																													
D38 .Number of PLHIV in care on First line regimen who had adverse events this month *(Essential Changes register)*																													
D39. Number of PLHIV in care on First line regimen who stopped treatment due to severe adverse events this month *(Essential Changes register)*																													
D40. Number of PLHIV in care on First line substituting due to toxicity this month* *(Essential Changes register)*																													
D41. Total number of PLHIV in care currently receiving first line ART (including those initiated during this current month). *(ART register)*																													

ART SUMMARY

AGE	≤ 2 months		3-12 months		13-24 months		25-59 months		5-9 years		10-14 years		15-19 years		20-24 years		25-29 years		30-34 years		35-39 years		40-44 years		45-49 years		50 years and above		Total
SEX	M	F	M	F	M	F	M	F	M	F	M	F	M	F	M	F	M	F	M	F	M	F	M	F	M	F	M	F	
D42.Number of PLHIV in care who died whilst on Second line ART regimen this month *(Essential Changes register)*																													
D43.Number of PLHIV lost to follow up whilst on Second line regimen this month. *(Essential Changes register)*																													
D44.Number of PLHIV in care who stopped treatment whilst on Second line regimen this month *(Essential Changes register)*																													
D45.Number of PLHIV in care who transferred out whilst on Second line regimen this month. *(Essential Changes register)*																													
D46.Number of PLHIV in care who transferred in whilst on Second line regimen this month. *(Essential Changes register)*																													
D47.Number of PLHIV in care on Second Line regimen who developed adverse events this month. *(Essential Changes register)*																													
D48.Number of PLHIV in care on Second Line regimen who have stopped treatment due to adverse events this month. *(Essential Changes register)*																													
D49. Total number of PLHIV in care currently receiving Second line ART (including those switched during the current month). *(ART register)*																													

N.B. Please ensure all adverse events/toxicities due to ARVs are notified to the Head of Station within 48 hours and appropriate Adverse Drug Reaction (ADR) forms are completed and sent to MCAZ through the institution's Pharmacy Manager

ART SUMMARY

AGE	≤2 months		3-12 months		13-24 months		25-59 months		5-9 years		10-14 years		15-19 years		20-24 years		25-29 years		30-34 years		35-39 years		40-44 years		45-49 years		50 years and above		Total
Sex	M	F	M	F	M	F	M	F	M	F	M	F	M	F	M	F	M	F	M	F	M	F	M	F	M	F	M	F	
D50.Number of PLHIV in care who died whilst on Third line ART regimen this month *(Essential Changes Register)*																											
D51.Number of PLHIV lost to follow up whilst on Third line regimen this month. *(Essential Changes Register)*																											
D52.Number of PLHIV in care who stopped treatment whilst on Third line regimen this month *(Essential Changes register)*																											
D53.Number of PLHIV in care transferred out whilst on Third line regimen this month. *(Essential Changes register)*																											
D54.Number of PLHIV in care transferred in on Third line regimen this month. *(Essential Changes Register)*																											
D55.Number of PLHIV in care on Third Line regimen who developed adverse events this month. *(Essential Changes register)*																											
D56.Number of PLHIV in care on Third Line ART regimen who had stopped due to adverse events this month. *(Essential changes register)*																											
D57. Total number of PLHIV in care currently receiving Third line ART(including those who had switched during the current month). *(ART Register)*																											
D58. Total number of PLHIV in care currently receiving ART this month*. *(Art Register)*																											
N.B. Add row D41, D49 and D57 to get the total number of PLHIV currently receiving ART in row D58.																											

N.B.Please ensure all adverse events/toxicities due to ARVs are notified to the Head of Station within 48 hours and appropriate Adverse Drug Reaction (ADR) forms are completed and sent to MCAZ through the institution's Pharmacy Manager.

E. WORKPLACE POST EXPOSURE PROPHYLAXIS (PEP)

	NEW			TO DATE		
SEX	M	F	Total	M	F	Total
E1. Total number of Health Care Workers who had an occupational HIV exposure this month. (Occupational Exposure Register)						
E2. Total number of Health Care Workers who accessed Post Exposure Prophylaxis (PEP) for HIV this month. (Occupational Exposure Register)						
E3. Total number of Health Care Workers who completed PEP this month. (Occupational Exposure Register)						
E4. Number Health Care Workers who sero- converted at 3 months post exposure, this month. (Occupational Exposure Register)						
E5. NumberHealthCareWorkerswhosero-convertedat6monthspostexposurethismonth. (Occupational Exposure Register)						

F.STI & SYPHILIS COLLABORATION

AGE	<=2 months		3 -12 months		1 3 -24 months		25 -59 months		5 -9 years		10 -14 years		15 -19 years		20 -24 years		25 -29 years		30 -34 years		35-39 years		40-44 years		45-49 years		50 + years		
SEX	M	F	M	F	M	F	M	F	M	F	M	F	M	F	M	F	M	F	M	F	M	F	M	F	M	F	M	F	Total
F1. Total number of new STI clients this month (STI Register)																													
F2. Total number of repeat STI clients this month (STI Register)																													
F3. Total number of STI clients who were tested for Syphilis this month (STI Register)																													
F4. Total number of STI clients who tested positive for Syphilis this month (STI Register)																													

F. STI INTEGRATION WITH HIV

	<=2 months		3-12 months		13-24 months		25-59 months		5-9 years		10-14 years		15-19 years		20-24 years		25-29 years		30-34 years		35-39 years		40-44 years		45-49 years		50+ years		Total
**SEX**	M	F	M	F	M	F	M	F	M	F	M	F	M	F	M	F	M	F	M	F	M	F	M	F	M	F	M	F	
F5.Total number of STI Clients HIV tested this month. *STI register*																													
F6.Total number of STI Clients Tested HIV positive this month *STI register*																													
F7.Total number of STI clients presenting with a known HIV positive status this month-*STI register*																													
F8.Number of clients given information on condom use. *STI Register*																													
F9.Total number of STI contacts treated this month *STI Register*																													

F. SEXUAL VIOLENCE

**AGE**	<=2 months		3-12 months		13-24 months		25-59 months		5-9 years		10-14 years		15-19 years		20-24 years		25-29 years		30-34 years		35-39 years		40-44 years		45-49 years		50+ years		Total
**SEX**	M	F	M	F	M	F	M	F	M	F	M	F	M	F	M	F	M	F	M	F	M	F	M	F	M	F	M	F	
F10.Total number of Clients Sexually Abused this month *(Medical Affidavit for sexual abuse register)*																													
F11.Total number of sexually abused clients reporting within 72 hours *(Medical Affidavit for sexual abuse register)*																													
F12.Total number of sexually abused clients received HIV counselling (Medical Affidavit for sexual abuse register)																													
F13.Total number sexually abused HIV tested this month *(Medical Affidavit for sexual abuse register)*																													
F14.Total number of sexually abused clients tested HIV positive this month *(Medical Affidavit for sexual abuse register)*																													
F15.Total number of sexually abused clients who received PEP for HIV this month. *(Medical Affidavit for sexual abuse register)*																													
F16. Total number of sexually abused clients who received PEP for STI this month*(Medical Affidavit for sexual abuse register)*																													
F17. Total number of sexually abused clients who received PEP for Pregnancy within 5 days this month*(Medical Affidavit for sexual abuse register)*																													

VOLUNTARY MEDICAL MALE CIRCUMCISION (VMMC)

AGE	<=2 months	3-12 months	13-24 months	25-59 months	5-9 years	10-12 years	13-14 years	15-19 years	20-24 years	25-29 years	30-34 years	35-39 years	40-44 years	45-49 years	50+ years	Total
G1.Number of HIV negative clients circumcised this month *(VMMC Client Register)*																
G2.Number of HIV positive clients circumcised this month *(VMMC Client Register)*																
G3.Number of clients with unknown HIV status circumcised this month *(VMMC Client Register)*																
G4.Total number of clients circumcised this month *(VMMC Client Register)*																
G5.Number of clients circumcised using dorsal slit method this month *(VMMC Client Register)*																
G6.Number of clients circumcised using forceps guided method this month *(VMMC Client Register)*																
G7.Number of clients circumcised using PrePex device this month *(VMMC Client Register)*																
G8. Total number of PrePex devices Used by size this month (not age related) *(VMMC Client Register)*	Size A			Size B					Size C			Size D		Size E		
G9.Number of clients experiencing at least one MODERATE adverse event (AE) during or following circumcision, this month *(VMMC Client Register)*																
G10.Number of clients experiencing at least one SEVERE adverse event (AE) during or following circumcision, this month *(VMMC Client Register)*																
G11.Total number of Moderate and Severe Adverse Events by TYPE** (not age related) this month *(VMMC Client Register*	**AN**	**BL**	**IN**	**PA**	**SD**	**SX**	**WD**	**DD**	**DH**	**DM**	**DP**	**DR**	**OA**			
G12. Number of clients who return at least once for post- operative follow-up care (routine or emergent) within 14 days of circumcision this month *(VMMC Client Register)*																
G13. Number of circumcised clients who received adequate TT vaccine this month *(VMMC Client Register)*																

***Adverse event type: AN Anaesthesia related problem; BL Bleeding; IN Infection; PA Pain; SD (Scarring/disfigurement/ poor cosmetic result; torsion; insufficient skin removal; excess skin removal; injury to penis;) SX (Sexual effects/ Undesirable sensory changes/dysfunction) WD Wound disruption; DD (Device Displacement/Detachment/Self removal) DH Delayed healing; DM Device malfunction; DP Difficult with placement; DR Difficult with device removal; OA: Other AEs, Excess swelling of penis/scrotum including haematoma; difficulty urinating*

**DD is only for devices*

H. CERVICAL CANCER SCREENING

	Type of Test	< 15 years	15-19 years	20-24 years	25-29 years	30-34 years	35-39 years	40-44 years	45-49 years	50+ years	Total
H1. Number of HIV-positive women on ART screened for cervical cancer *(Cervical Cancer register)*											
H2. Number of HIV-positive women on ART screened for cervical cancer for the "first time" this month *(Cervical Cancer register)*											
H3. Number of HIV-positive women on ART screened for cervical cancer for "post treatment follow-up screening" this month *(Cervical Cancer register)*											
H4. Total number of HIV positive women on ART screened for cervical cancer with "negative" VIAC result this month. *(Cervical Cancer register)*	a. First Time										
	b. Rescreen										
	c. Post-Treatment Follow-up										
H5. Total number of HIV positive women on ART screened for cervical cancer with "positive" VIAC result this month. *(Cervical Cancer register)*	a. First Time										
	b. Rescreen										
	c. Post-Treatment Follow-up										
H6. Total number of HIV positive women on ART screened for cervical cancer with "suspected cancer" VIAC result this month. *(Cervical Cancer register)*	a. First Time										
	b. Rescreen										
	c. Post-Treatment Follow-up										
H7. Total number of HIV positive women on ART screened for cervical cancer with "positive" VIAC result, who are eligible for cryotherapy, thermocoagulation or LEEP this month. *(Cervical Cancer register)*	a. First Time										
	b. Rescreen										
	c. Post-Treatment Follow-up										
H8. Total number of HIV positive women on ART screened for cervical cancer with "positive" VIAC result, who are eligible for and received cryotherapy this month.	a. First Time										
	b. Rescreen										
	c. Post-Treatment Follow-up										
H9. Total number of HIV positive women on ART screened for cervical cancer with "positive" VIAC result, who are eligible for and received thermocoagulation this month.	a. First Time										
	b. Rescreen										
	c. Post-Treatment Follow-up										
H10. Total number of HIV positive women on ART screened for cervical cancer with "positive" VIAC result, who are eligible for and received LEEP this month.	a. First Time										
	b. Rescreen										
	c. Post-Treatment Follow-up										

Comments……………………………………………………………………………………
