# Supplementary material for: Impact of the COVID-19 pandemic on the provision and uptake of services for the prevention of mother-to-child transmission of HIV in Zimbabwe
Source: PLOS Glob Public Health. 2023 Aug 14;3(8):e0002296. doi: 10.1371/journal.pgph.0002296 (PMC10424857; doi:10.1371/journal.pgph.0002296)
Supplement: Attachment — Submitted filename: UC MoHCC paper - response to reviewer comments.docx [file pgph.0002296.s017.docx]

**Manuscript reference number: PGPH-D-22-02047**

Dear Editor,

We are resubmitting a revised version of our manuscript **PGPH-D-22-02047**, titled "Impact of the COVID-19 pandemic on the provision and uptake of services for the prevention of mother-to-child transmission of HIV in Zimbabwe” in line with the comments received.

We appreciate the reviewers time and their valuable comments and suggestions. We have addressed each comment point-by-point in the response below and have amended the manuscript accordingly. We have also amended the Financial Disclosure statement as requested.

Thank you for considering our work.

Sincerely,

Lizzie Chappell on behalf of the co-authors.

**Response to the reviewers**

Reviewer #1: The authors present a Impact of the COVID-19 pandemic on the provision and uptake of services for the
prevention of mother-to-child transmission of HIV in Zimbabwe. The paper is well written, informative, novel and contains plenty of data. This reviewer is in favor of accepting this manuscript for publication

Reviewer #2: Excellent paper.
Please make a few minor edits:
1. In Paragraph 1 of the introduction, use past instead of present tense where appropriate

Response: We have amended this paragraph as suggested.

2. In Table 1 - for denominators, please use "estimated number" instead of "number" where appropriate
3. Please do the same for the results narrative as well - be very clear in regards to what are "estimated number of" and "estimated proportion of" vs. absolute counts.

**Response:**

**We have updated the manuscript as suggested. We all refer to all numbers from ZimStat and Spectrum as ‘estimated numbers’, but not those from DHIS-2 as these are actual counts of facility activity. All proportions are referred to as ‘estimated proportions’.**
